# A Novel Artificial Bee Colony Based Clustering Algorithm for Categorical Data

**DOI:** 10.1371/journal.pone.0127125

**Published:** 2015-05-20

**Authors:** Jinchao Ji, Wei Pang, Yanlin Zheng, Zhe Wang, Zhiqiang Ma

**Affiliations:** 1 School of Computer Science and Information Technology, Northeast Normal University, Changchun, China; 2 Key Lab of Intelligent Information Processing of Jilin Universities, Northeast Normal University, Changchun, China; 3 Key Laboratory of Symbolic Computation and Knowledge Engineering of Ministry of Education, Jilin University, Changchun, China; 4 School of Natural and Computing Sciences, University of Aberdeen, Aberdeen, United Kingdom; 5 College of Computer Science and Technology, Jilin University, Changchun, China; Shenzhen Institutes of Advanced Technology, CHINA

## Abstract

Data with categorical attributes are ubiquitous in the real world. However, existing partitional clustering algorithms for categorical data are prone to fall into local optima. To address this issue, in this paper we propose a novel clustering algorithm, ABC-K-Modes (Artificial Bee Colony clustering based on K-Modes), based on the traditional k-modes clustering algorithm and the artificial bee colony approach. In our approach, we first introduce a one-step k-modes procedure, and then integrate this procedure with the artificial bee colony approach to deal with categorical data. In the search process performed by scout bees, we adopt the multi-source search inspired by the idea of batch processing to accelerate the convergence of ABC-K-Modes. The performance of ABC-K-Modes is evaluated by a series of experiments in comparison with that of the other popular algorithms for categorical data.

## Introduction

As an important technique in data mining, clustering analysis has been used in many fields [1,2], such as information retrieval [3], social media analysis [4], privacy preserving [5], image analysis [6], text analysis [7], and bioinformatics [8]. The aim of clustering is to group those data objects with similar characteristics into the same clusters, and the ones with dissimilar characteristics into different clusters. Most existing clustering algorithms in the literature belong to one of the following two types: hierarchical and partitional. Hierarchical clustering algorithms allocate a group of data objects into a dendrogram of the nested partitions according to a divisive or agglomerative strategy [9]. While partitional clustering algorithms partition a set of data objects into a pre-defined number of clusters by optimizing an objective cost function.

Center-based clustering algorithms are the most popular partitional clustering algorithms. The k-means algorithm is a widely used center-based partitional clustering algorithm due to its simplicity and high efficiency [10]. Considering the uncertainty of data objects, the fuzzy k-means algorithm [11] is also developed. The k-means algorithm and the fuzzy k-means algorithm can only deal with numeric data. However, categorical data are frequently encountered in real world applications, and especially in the emerging social media analysis. For instance, clustering Twitter users based on their profiles described by categorical attributes. For clustering categorical data, Huang extended these two classical algorithms and introduced the well-known k-modes algorithm and fuzzy k-modes algorithm [12–14]. However, one issue associated with (fuzzy) k-means and (fuzzy) k-modes algorithms is that they may fall into local optima. To address this issue, many heuristic clustering algorithms, which adopt the optimization procedures in the clustering process, have been proposed. By introducing genetic algorithms (GAs), the GA-based clustering approaches [15], including the genetic k-means algorithm [16], the fast genetic k-means algorithm [17], and the genetic k-modes algorithm [18] have been developed. Among these GA-based clustering algorithms, the genetic k-modes algorithm [18] is suitable for categorical data. In addition, the following heuristic clustering algorithms are used to cluster numeric data: Selim and Al-Sultan introduced a simulated annealing algorithm for the clustering problem [19]. Maulik and Mukhopadhyay introduced a novel fuzzy clustering approach by integrating the simulated annealing heuristic with artificial neural networks [20]. Sung and Jin presented a tabu search-based clustering approach by combining the packing and releasing procedures [21].

Over the last decade, a few approaches have been developed to model the intelligent foraging behavior of social animals, such as birds and ants, for optimization problems, and these approaches have been successfully applied to clustering. Shelokar, Jayaraman, and Kulkarni proposed an ant colony clustering algorithm which simulates the way real ants look for an optimal path from their nest to a food source [22]. Kao, Zahara, and Kao integrated the particle swarm optimization (PSO) approach, which mimics the way birds find the optimal food sources in search space, with the k-means procedure and Nelder–Mead simplex search method for improving the performance of clustering [23]. Unlike Kao's approach, Tunchan proposed a pure PSO approach for clustering [24]. Chuang, Hsiao, and Yang presented an accelerated chaotic map particle swarm optimization (ACPSO) for clustering by integrating the chaotic map particle swarm optimization (CPSO) with an accelerated convergence rate strategy [25]. Wan *et al*. introduced a clustering algorithm on the basis of the optimization property of bacterial foraging behavior [26].

In recent years, investigating the foraging behavior of honeybees, including the learning, memorising, and information sharing mechanism, has emerged as an interesting research direction in swarm intelligence [27]. Inspired by the foraging behavior of bee swarms in the real world, Lucic and Teodorović introduced the bee colony optimization heuristic [28], which has been used for solving various engineering and management problems. Karaboga and Basturk presented an artificial bee colony (ABC) algorithm [29] to deal with numerical optimization problems. By using the ABC optimization strategy, Karaboga and Ozturk proposed an artificial bee colony clustering approach [30]. Almost at the same time, Zhang, Ouyang and Ning also introduced an artificial bee colony clustering approach, in which Deb’s rules were used to direct the search direction of each candidate food source [27]. However, most of these heuristic approaches are designed for numeric data, and therefore they are not suitable to deal with categorical data. Considering the ubiquity of categorical data in real-world applications, it is necessary to develop an ABC-based clustering algorithm for categorical data.

In this paper, we propose a novel artificial bee colony clustering approach for categorical data. In our approach, we first introduce the one-step k-modes procedure, and then integrate this procedure with the artificial bee colony heuristic to cluster categorical data. The time and space complexity of the proposed approach is analysed, and a comparison with the other popular approaches demonstrates the effectiveness of our approach.

The remainder of this paper is organised as follows: we first review some related work. This is followed by the presentation of our proposed method. Then, we report the experimental results, which demonstrate the advantages of the proposed method. Finally, we draw conclusions and explore future work.

## Related Work

In this section, we first review the k-modes algorithm, and then describe the idea of artificial bee colony optimization.

### The k-modes algorithm

The k-modes algorithm was first introduced by Huang in [31] for clustering categorical data. Let *X* = {*x*_1_, *x*_2_,…,*x*_*n*_} denote a dataset consisting of *n* data objects and *x*_*i*_ (1 ≤ *i* ≤ *n*) be a data object characterised by *m* categorical attributes *A*_1_, *A*_2_,…, *A*_*m*_. Each categorical attribute *A*_*j*_ has a domain of values denoted by $Dom(A_{j})=\{a_{j}^{1},a_{j}^{2},\ldots ,a_{j}^{t}\}$, where *t* is the number of categorical values for the attribute *A*_*j*_. A data object *x*_*i*_ is generally represented in the form of a vector [*x*_*i*1_, *x*_*i*2_,…, *x*_*im*_].

The aim of the k-modes algorithm is to divide a dataset *X* into *k* clusters by minimizing the following cost function:

$$E(U,Q)=\sum_{l=1}^{k}\sum_{i=1}^{n}u_{il}dis(x_{i},Q_{l}).$$ (1)

Here *Q*_*l*_ is the set of the most frequent value for each attribute in a cluster *l*, and it is called the mode of the cluster *l*; *u*_*il*_ (0 ≤ *u*_*il*_ ≤ 1) is an element of the partition matrix *U*_*n*×*k*_; *k* is the number of clusters, and *dis*(*x*_*i*_, *Q*_*l*_) is the distance measure as given below:

$$dis\left(x_{i},Q_{l}\right)=\sum_{j=1}^{m}\alpha \left(x_{ij},q_{lj}\right).$$ (2)

In Eq (2), *α*(*x*_*ij*_, *q*_*lj*_) is defined as:

$$\alpha (x_{ij},q_{lj})=\left\{ \begin{array}{l} 0,\;\text{if}\;x_{ij}=q_{lj},\\ 1,\;\text{if}\;x_{ij}\neq q_{lj}, \end{array}\right.$$ (3)

where *q*_*lj*_ is the most frequent value of the *jth* categorical attribute in the cluster *l*. The process of the k-modes algorithm is depicted as follows:

*Step 1.* Randomly pick up *k* data objects from the dataset *X* as the initial modes of clusters.
*Step 2.* For each data object in *X*, assign it to the cluster the mode of which is the nearest one to this data object compared to the modes of other clusters in terms of Eq (2). After all data objects have been assigned to clusters, update the modes of all clusters.
*Step 3.* Re-evaluate the dissimilarity between the data objects and the current modes after all data objects have been assigned to clusters. If it is found that a data object’s nearest mode belongs to another cluster rather than the current one, reassign this data object to that cluster and update the modes of both clusters.
*Step 4.* After a full circle test of *X*, if no data objects have changed clusters, terminate the algorithm; otherwise go to Step 3.

### The artificial bee colony algorithm

The artificial bee colony (ABC) algorithm proposed by Karaboga and Basturk [29] is well-known for its simplicity and robustness for optimising numeric problems. In the ABC algorithm, the artificial bee swarm consists of three types of bees: employed bees, onlookers, and scouts. The employed bee takes a particular food source to exploit and shares the information about the food source with onlookers in the nest; a scout looks for a new food source in the search space, and an onlooker waits in the nest and finds a food source through the information shared by employed bees. The artificial bee colony has two parts: the first half are the employed bees and the second half are the onlookers. In the model of forage selection, three essential components (food sources, employed foragers, and unemployed foragers) and two modes of the behavior (recruitment to a food source and abandonment of a food source) are given. The value of food source is associated with many factors such as its proximity to the nest, nectar amount and the ease of gathering this nectar. The unemployed forgers contain two types of bees: scouts and onlookers. There is only one employed bee on a food source. Thus, the number of employed bees is equal to the number of food sources. Onlookers move onto a food source according to a probability-based selection strategy. When the nectar of a food source is exhausted, the corresponding employed bee becomes a scout. In ABC algorithm, the exploitation and exploration processes are performed together. Specifically, the employed bees and onlookers implement the exploitation process, and the scouts execute the exploration process. The bee colony explores and exploits the food sources in a way to maximize the nectar being stored in the nest. For an optimisation problem, a food source means a possible solution, the nectar amount of a food source measures the quality of the corresponding solution, and the goal is to obtain the optimal value of the objective function. The procedure of ABC algorithm is given as follows:

*Step 1.* Initialize the population of food sources.
*Step 2.* Send the employed bees onto the food sources and evaluate the corresponding nectar amounts.
*Step 3.* Evaluate the probabilities of all food sources to be chosen by the onlooker bees, and the probability value of each food source is determined by its nectar amount (i.e., the quality of the corresponding solution): the bigger the nectar amount of the food source, the higher the probability value is;
*Step 4.* Send the onlookers onto the food sources: each onlooker will chose its food source based on the probabilities calculated from Step 3, exploit its food source, evaluate the nectar amount of the obtained food source, and apply greedy selection process;
*Step 5.* Terminate the exploitation process of an employed bee if its food source becomes exhausted, and this employed bee becomes a scout bee;
*Step 6.* Send the scouts into the search space for finding new food sources randomly;
*Step 7.* Memorise the best food source found so far;
*Step 8.* If the requirements are met, output the best food source; otherwise go to Step 2.

## Our Proposed ABC Clustering Algorithm

In this section, we first describe our proposed ABC clustering approach, and then discuss the complexity and convergence of this approach.

### The proposed approach

In this subsection, we propose a novel clustering algorithm on the basis of artificial bee colony and the k-modes approach. As mentioned above, there are three types of artificial honeybees: employed bees, onlookers, and scouts. A food source corresponds to a possible solution of the problem to be optimised, and the nectar amount of a food source characterises the quality of the corresponding solution. In the clustering, the clustering results depend on the cluster centers. When the cluster centers are fixed, the clustering results are determined. Therefore, the clustering issue can be seen as the optimisation of the cluster centers, and a set of cluster centers correspond a possible solution. For categorical data clustering, let *f*_*i*_ = {Q_1_, Q_2_,…, Q_*k*_} denote a food source, where *Q*_*l*_ is the mode of cluster *l*. *E*(*f*_*i*_) = *E*(*U*, *f*_*i*_) is the objective cost function, and $E(U,f_{i})=\sum_{l=1}^{k}\sum_{i=1}^{n}u_{il}dis(x_{i},Q_{l})$, where the symbols have the same meaning as in Eq (1). Then, the nectar amount of a food source *f*_*i*_ is given by:

$$NA(f_{i})=\frac{1}{E(f_{i})+1}$$ (4)

Similar to the ABC approach, the colony of artificial bees in our algorithm has two parts: the first half of the artificial bees are the employed bees, and the second half of the artificial bees are the onlookers. There exists only one employed bee for a food source, and the number of the employed bees is equal to the number of solutions in the population. Let *P*_*fs*_ = {*f*_1_, *f*_2_,…, *f*_*H*_} denote the population of food sources, where *H* is the number of the food sources, and *f*_*i*_ is the *i*th food source. Then the probability of the *i*th food source being picked up by an onlooker is given by:

$$pro_{i}=\frac{NA(f_{i})}{\sum_{j=1}^{H}NA(f_{j})}$$ (5)

For deriving a candidate food source from the current one in memory, we introduce the one-step k-modes procedure, called OKM, in our algorithm. The OKM procedure is essentially one iteration step in the search process of the k-modes algorithm, and it is used to search the neighbor food source based on the current food source in the exploitation process performed by employed bees and onlookers. Let *f*_*i*_ be the current food source, then the OKM consists of the following two steps:

1. Allocate each data object to the cluster with the nearest mode, and then form a partition matrix *U*; specifically, if the *i*th data object belongs to the *l*th cluster *u*_*il*_ = 1; otherwise *u*_*il*_ = 0, where *u*_*il*_ is one element of *U*;
2. Calculate the new modes on the basis of the partition matrix *U*, and thus form a candidate food source fi'={Q1',Q2',…,Qk'}.

For the colony of bees, an employed bee becomes a scout when its food source is exhausted. In our algorithm we adopt the parameter *L*, which is a predetermined number of trials to control the abandonment of a food resource. If a food source cannot be improved further through *L* trials, this food source is assumed to be abandoned, and the corresponding employed bee becomes a scout. Let the abandoned food source be *f*_*i*_, and then the search operation of a scout finding a new food source is given by:

fi'=Rand(Dom(X)), (6)

where *i* ∈ {1, 2,…, *H*}, and *Rand*(*Dom*(*X*)) is the operation of randomly selecting *k* data objects from the data set *X*. In our algorithm, the multi-source search, which is inspired by the idea of batch processing [32], is adopted to accelerate the convergence of the proposed algorithm. The idea of the multi-source search is described as follows: a scout bee searches *T* candidate food sources at a time, and then picks up the best one as the new food source.

Having introduced the detailed calculation formula for relevant variables, the proposed ABC-K-Modes clustering algorithm for categorical data is given as follows:

*Input*: The size of bee colony *N*, the maximum cycle number *MCN*, the number of clusters *k*, and *L*.

*Output*: The best food source.

1. Initialise the population of food sources *P*_*fs*_ = {*f*_1_, *f*_2_,…, *f*_*H*_} randomly; specifically, for each food source, select *k* data objects randomly from the dataset *X* as the modes of clusters; set the exploitation numbers of food sources *En*_*1*_ *= 0*, *En*_*2*_ *= 0*,…, *En*_*H*_ *= 0*.
2. Evaluate the nectar amounts of the food sources *NA(f*_*1*_ *)*, *NA(f*_*2*_ *)*, *… NA(f*_*H*_ *)*, according to Eq (4);
3. set *CN*(the cycles number) to 1;
4. For each employed bee
  - Generate a new food source *f*_*i*_' from the current food source *f*_*i*_ by using the one-step k-modes procedure OKM, and set *En*_*i*_ *= En*_*i*_ *+*1;
  - Evaluate the nectar amount *NA*(*f*_*i*_') for the food source *f*_*i*_' according to Eq (4);
  - If *NA*(*f*_*i*_') > *NA*(*f*_*i*_), the current food source *f*_*i*_ is replaced by the new food source; otherwise the current food source *f*_*i*_ is retained.
5. Evaluate the probability *pro*_*i*_ for each food source *f*_*i*_ according to Eq (5);
6. For each onlooker bee
  - Pick up one food source *f*_*i*_ as the current food source according to the calculated probabilities;
  - Generate a new food source *f*_*i*_' from the current food source *f*_*i*_ by using OKM, and set *En*_*i*_ *= En*_*i*_ *+*1;
  - Evaluate the nectar amount of *f*_*i*_', that is, *NA*(*f*_*i*_');
  - If *NA*(*f*_*i*_') > *NA*(*f*_*i*_), the current food source *f*_*i*_ is replaced by the new food source *f*_*i*_'; otherwise the current food source *f*_*i*_ is retained;
  - Update the probability *pro*_*i*_ for each food source *f*_*i*_ according to Eq (5).
7. For each food source *f*_*i*_, if the exploitation number *En*_*i*_ is no less than *L*, this food source is abandoned, and the corresponding employed bee becomes a scout.
8. If there exists an abandoned food source *f*_*i*_,
  - Send the scout in the search space to find *T* candidate food sources $\left\{f_{i}^{1},f_{i}^{2},\ldots ,f_{i}^{T}\right\}$ according to Eq (6);
  - Evaluate the nectar amounts $\left\{NA(f_{i}^{1}),NA(f_{i}^{2}),\ldots ,NA(f_{i}^{T})\right\}$ of the food sources $\left\{f_{i}^{1},f_{i}^{2},\ldots ,f_{i}^{T}\right\}$;
  - Choose the food source with the highest nectar amount as the new food source *f*_*i*_', and set *En*_*i*_ *=* 0;
  - If *NA*(*f*_*i*_') > *NA*(*f*_*i*_) the current food source *f*_*i*_ is replaced by the new food source *f*_*i*_'; otherwise the current food source *f*_*i*_ is retained.
9. *CN = CN+1*;
10. If *CN = MCN*, terminate the algorithm and output the best food source; otherwise go to step 4).

### Complexity analysis

In this subsection, we discuss the complexity of the proposed ABC-K-Modes approach. The time complexity of the proposed method mainly consists of five parts: the initialisation, the search operation of employed bees, the calculation of the probability of food sources, and the search operation of scouts and onlookers. The computational cost of these five parts are *O*(*Hknm*), *O*(*H*(*nkm* + *nkC*)), *O*(*H*), *O*(*Tnkm*), and *O*(*H*(*H* + (*nkm* + *nkC*) + *nkm*)), respectively. Here *n* is the number of data objects in the dataset *X*; *m* is the number of attributes; *k* is the number of clusters; *H* is the number of employed bees or food sources; $C=\sum_{j=1}^{m}t_{i}$ is the total number of categories for all attributes. Therefore, the overall time complexity of the proposed approach is *O*(*Hkmn* + *s*(*Tnkm* + *H*(*H* + *nkm* + *nkC*))). Here, *s* is the number of iterations. For space complexity, it requires *O*(*mn*) to store the dataset *X*, *O*((*H* + *T*)*km*) to store the food sources, and *O*(*nk*) to store the partition matrix. Thus, the overall space complexity of our method is *O*(*mn* + (*H* + *T*)*km* + *nk*). The time complexity and the space complexity of the k-modes algorithm are *O*(*nkm* + *s*(*nkC* + *nkm*)) and *O*(*m*(*n* + *k*) + *km*), respectively. For genetic k-modes algorithm, the time complexity and space complexity are *O*(*Nn* + *S*(*N* ^2^ *nkm* + *N*(*n* ^2^ *kC* + *nkm*) + *nkC* + *nkm*)) and *O*(*mn* + *Hn* + *Hkm*), respectively. Here *N* is the size of population, and *S* is the maximal number of generations. Generally, when *H*, *m*, *k* << *n*, the complexity of our algorithm is higher than k-modes algorithm, and lower than genetic k-modes algorithm.

### Convergence analysis

In this subsection, we discuss the convergence of the proposed approach. In our approach, the exploration and exploitation are both executed by ABC. For a categorical dataset, the number of different values for an attribute is finite, and the number of attributes is finite as well. It is noted that a candidate solution is a set of cluster centers, and a cluster center is a set of attributes values. Therefore, the number of candidate solutions is finite. Specifically, the number of candidate solutions is $\left(\begin{array}{l} L\\ k \end{array}\right)$, where $L=\left(\begin{array}{l} \left|A_{1}\right|\\ 1 \end{array}\right)\times \left(\begin{array}{l} \left|A_{2}\right|\\ 1 \end{array}\right)\times \cdots \times \left(\begin{array}{l} \left|A_{m}\right|\\ 1 \end{array}\right)$, and *k* is the number of clusters. Here, |*A*_*i*_| is the number of different categorical values for the attribute *A*_*i*_. In the process of exploration or exploitation, the current solution will be replaced by a new solution if the new one is better. Thus each possible solution appears at most once in the current solution list. If the value of MCN (maximum number of iterations) is large enough, the global optimal solution will be very likely to be found; otherwise, the algorithm will be converged to a local optimum. In other words, the larger the value of MCN, the greater the possibility that ABC-K-Modes will converge is. When MCN tends to be infinite, the possibility of convergence for our proposed approach approaches to 100%. Therefore the convergence of our algorithm to a global/local optimal solution is guaranteed as long as MCN is big enough. However, due to different characteristics of the search spaces to be explored, for each dataset a different value of MCN may be required for the algorithm to converge.

## Experimental Results and Discussion

In this section, for evaluating the performance of our proposed clustering algorithm ABC-K-Modes, we run the proposed approach on six real-world categorical datasets: Zoo, Breast cancer, Soybean, Lung cancer, Mushroom, and Dermatology, all of which can be downloaded from UCI Machine Learning Repository (http://archive.ics.uci.edu/ml/datasets.html). In this research, we adopt Yang’s accuracy measure [33] and the Rand Index [34] to assess the obtained clustering results. In Yang's method, the definitions of accuracy (*AC*), precision (*PR*), and recall (*RE*) are given as follows:

$$AC=\frac{\sum_{i=1}^{k}a_{i}}{n},$$ (7)

$$PR=\frac{\sum_{i=1}^{k}\frac{a_{i}}{a_{i}+b_{i}}}{k},$$ (8)

$$RE=\frac{\sum_{i=1}^{k}\frac{a_{i}}{a_{i}+c_{i}}}{k},$$ (9)

where *a*_*i*_ is the number of data objects that are correctly allocated to class *C*_*i*_, *b*_*i*_ is the number of data objects that are incorrectly allocated to class *C*_*i*_, *c*_*i*_ is the number of data objects that are incorrectly denied from class *C*_*i*_, *k* is the total number of classes contained in a dataset, and *n* is the total number of data objects in a dataset. In the above measures, the AC has the same meaning as the clustering accuracy *r* defined in [12]. Given a dataset *X* = {*x*_1_, *x*_2_,…, *x*_*n*_} as well as two partitions of this dataset: $Y=\{{\text{y}}_{1},{\text{y}}_{2},\ldots ,{\text{y}}_{t_{1}}\}$ and Y'={y1',y2',…,yt2'}, the Rand Index (*RI*) [34] is given by

$$RI=\frac{\sum_{i=1,j=2;i<j}^{n}\alpha_{ij}}{\left(\begin{array}{l} n\\ 2 \end{array}\right)},$$ (10)

where

αij={1,if there existtandt'such that bothxiandxjare in both ytandyt'',1,if there existtandt'such thatxiis in both ytandyt''whilexjis in neither ytoryt'',0,otherwise. (11)

The *RI* is calculated by using the true clustering and the clustering obtained from a clustering algorithm. According to these measures, the higher values of *AC*, *PR*, *RE*, and *RI* indicate a better clustering result. In the performance analysis, we run our proposed ABC-K-Modes algorithm, the k-modes algorithm, the fuzzy k-modes algorithm, and the genetic k-modes approach on six different datasets, and for each dataset we run twenty trials. We then compare the clustering result of the proposed ABC-K-Modes algorithm with that of the other three well-known algorithms in terms of the best (Best), average (Avg.), and standard deviation (Std.) of *AC*, *PR*, *RE*, and *RI*, respectively. All algorithms are implemented in Java language and executed on an Intel(R) Core(TM) i7, 3.4GHz, 8GB RAM computer. In all experiments, the parameters of the proposed ABC-K-Modes algorithm are set as follows: *N =* 20, *MCN =* 1000, which are the typical values used in the original ABC algorithm [30]; *L =* 5 and *T =* 5 are set by the rule of thumb. The cluster number *k* in all four algorithms is set according to the number of classes provided by the class information of the dataset. We remark that other class information is not used in the clustering process apart from the number of classes. The other parameters of the k-modes algorithm, fuzzy k-modes algorithm, and the genetic k-modes are set the same as those stated in their original papers.

The Zoo dataset consists of 101 data objects, each of which has 17 Boolean-valued attributes. According to the class attributes, all data objects belong to one of the seven classes. Tables 1–4 list the comparison of clustering results of ABC-K-Modes, the k-modes, fuzzy k-modes, and the genetic k-modes on the Zoo dataset according to *AC*, *PR*, *RE*, and *RI*, respectively.

**Table 1:** The AC of the four algorithms on the Zoo dataset.

Algorithms	AC		
	Best	Avg.	Std.
**ABC-K-Modes**	0.9307	0.9134	0.0124
**K-modes**	0.9109	0.8287	0.0502
**Fuzzy k-modes**	0.9208	0.8371	0.0490
**Genetic k-modes**	0.9208	0.9074	0.0141

**Table 2:** The PR of the four algorithms on the Zoo dataset.

Algorithms	PR		
	Best	Avg.	Std.
**ABC-K-Modes**	0.9089	0.8796	0.0162
**K-modes**	0.8798	0.8331	0.0452
**Fuzzy k-modes**	0.8828	0.7761	0.0988
**Genetic k-modes**	0.8819	0.8694	0.0126

**Table 3:** The RE of the four algorithms on the Zoo dataset.

Algorithms	RE		
	Best	Avg.	Std.
**ABC-K-Modes**	0.8286	0.8144	0.0083
**K-modes**	0.8145	0.6126	0.1036
**Fuzzy k-modes**	0.8143	0.6375	0.1026
**Genetic k-modes**	0.8143	0.8024	0.0244

**Table 4:** The RI of the four algorithms on the Zoo dataset.

Algorithms	RI		
	Best	Avg.	Std.
**ABC-K-Modes**	0.9766	0.8969	0.0079
**K-modes**	0.9549	0.8758	0.0465
**Fuzzy k-modes**	0.9604	0.8787	0.0388
**Genetic k-modes**	0.9018	0.8934	0.0079

The Breast Cancer dataset contains 699 data objects, each of which is described by 10 categorical attributes. According to the class attribute, the data objects belong to one of the two classes: Benign and Malignant. Tables 5–8 summarise the comparison of the clustering results of ABC-K-Modes and the other three well-known algorithms on the Breast Cancer dataset according to *AC*, *PR*, *RE*, and *RI*, respectively.

**Table 5:** The AC of the four algorithms on the Breast Cancer dataset.

Algorithms	AC		
	Best	Avg.	Std.
**ABC-K-Modes**	0.9399	0.9199	0.0134
**K-modes**	0.9399	0.8568	0.1201
**Fuzzy k-modes**	0.9399	0.7694	0.1338
**Genetic k-Modes**	0.6552	0.6552	0.0000

**Table 6:** The PR of the four algorithms on the Breast Cancer dataset.

Algorithms	PR		
	Best	Avg.	Std.
**ABC-K-Modes**	0.9385	0.9320	0.0044
**K-modes**	0.9385	0.8785	0.0994
**Fuzzy k-modes**	0.9385	0.7988	0.1187
**Genetic k-Modes**	0.7439	0.7096	0.0480

**Table 7:** The RE of the four algorithms on the Breast Cancer dataset.

Algorithms	RE		
	Best	Avg.	Std.
**ABC-K-Modes**	0.9276	0.8924	0.0236
**K-modes**	0.9276	0.7998	0.1791
**Fuzzy k-modes**	0.9276	0.6768	0.2021
**Genetic k-Modes**	0.5000	0.5000	0.0000

**Table 8:** The RI of the four algorithms on the Breast Cancer dataset.

Algorithms	RI		
	Best	Avg.	Std.
**ABC-K-Modes**	0.8869	0.8527	0.0229
**K-modes**	0.8869	0.7744	0.1565
**Fuzzy k-modes**	0.8869	0.6571	0.1757
**Genetic k-modes**	0.5197	0.5178	0.0027

The Soybean dataset is composed of 47 data objects, each of which has 36 categorical attributes. In terms of the class attribute, the data objects belong to one of the four diseases: Diaporthe Stem Canker, Charcoal Rot, Rhizoctonia Root Rot, and Phytophthora Rot. Tables 9–12 list the comparison of the clustering results of ABC-K-Modes and the other three well-known algorithms on the Soybean dataset according to *AC*, *PR*, *RE*, and *RI*, respectively.

**Table 9:** The AC of the four algorithms on the Soybean dataset.

Algorithms	AC		
	Best	Avg.	Std.
**ABC-K-Modes**	1.0000	0.9862	0.0199
**K-modes**	1.0000	0.8883	0.1193
**Fuzzy k-modes**	1.0000	0.8032	0.1074
**Genetic k-modes**	1.0000	0.9829	0.0191

**Table 10:** The PR of the four algorithms on the Soybean dataset.

Algorithms	PR		
	Best	Avg.	Std.
**ABC-K-Modes**	1.0000	0.9864	0.0195
**K-modes**	1.0000	0.9409	0.0497
**Fuzzy k-modes**	1.0000	0.8419	0.1046
**Genetic k-modes**	1.0000	0.9829	0.0188

**Table 11:** The RE of the four algorithms on the Soybean dataset.

Algorithms	RE		
	Best	Avg.	Std.
**ABC-K-Modes**	1.0000	0.9904	0.0137
**K-modes**	1.0000	0.8765	0.1444
**Fuzzy k-modes**	1.0000	0.7728	0.1279
**Genetic k-modes**	1.0000	0.9882	0.0131

**Table 12:** The RI of the four algorithms on the Soybean dataset.

Algorithms	RI		
	Best	Avg.	Std.
**ABC-K-Modes**	1.0000	0.9850	0.0216
**K-modes**	1.0000	0.9023	0.1078
**Fuzzy k-modes**	1.0000	0.8507	0.0726
**Genetic k-modes**	1.0000	0.9813	0.0208

The Lung Cancer dataset has 32 data objects, each of which is described by 57 categorical attributes. According to the class attribute, the dataset has three classes. Tables 13–16 summarise the comparison of the clustering results of the ABC-K-Modes algorithm and the other three well-known algorithms on the Lung Cancer dataset according to *AC*, *PR*, *RE*, and *RI*, respectively.

**Table 13:** The AC of the four algorithms on the Lung Cancer dataset.

Algorithms	AC		
	Best	Avg.	Std.
**ABC-K-Modes**	0.6563	0.5578	0.0529
**K-modes**	0.5938	0.5344	0.0417
**Fuzzy k-modes**	0.5938	0.5313	0.0517
**Genetic k-modes**	0.6562	0.5562	0.0561

**Table 14:** The PR of the four algorithms on the Lung Cancer dataset.

Algorithms	PR		
	Best	Avg.	Std.
**ABC-K-Modes**	0.7152	0.6142	0.0662
**K-modes**	0.6955	0.5992	0.0790
**Fuzzy k-modes**	0.7033	0.5757	0.0880
**Genetic k-modes**	0.6905	0.5974	0.0783

**Table 15:** The RE of the four algorithms on the Lung Cancer dataset.

Algorithms	RE		
	Best	Avg.	Std.
**ABC-K-Modes**	0.6530	0.5654	0.0501
**K-modes**	0.6333	0.5390	0.0560
**Fuzzy k-modes**	0.6333	0.5504	0.0648
**Genetic k-modes**	0.6481	0.5619	0.0546

**Table 16:** The RI of the four algorithms on the Lung Cancer dataset.

Algorithms	RI		
	Best	Avg.	Std.
**ABC-K-Modes**	0.6593	0.6019	0.0270
**K-modes**	0.6431	0.5919	0.0294
**Fuzzy k-modes**	0.6331	0.5976	0.0241
**Genetic k-modes**	0.6452	0.6010	0.0260

Mushroom dataset contains 8,124 data objects, each of which has 23 categorical attributes. According to the class attribute, each data object falls into one of the two classes: edible and poisonous. Tables 17–20 list the comparison of the clustering results of ABC-K-Modes and the other three well-known algorithms on the Mushroom dataset according to *AC*, *PR*, *RE*, and *RI*, respectively.

**Table 17:** The AC of the four algorithms on the Mushroom dataset.

Algorithms	AC		
	Best	Avg.	Std.
**ABC-K-Modes**	0.8946	0.8573	0.0993
**K-modes**	0.8000	0.5998	0.0698
**Fuzzy k-modes**	0.8326	0.7033	0.0997
**Genetic k-modes**	0.6489	0.5523	0.0448

**Table 18:** The PR of the four algorithms on the Mushroom dataset.

Algorithms	PR		
	Best	Avg.	Std.
**ABC-K-Modes**	0.9128	0.8725	0.1047
**K-modes**	0.8574	0.6058	0.0874
**Fuzzy k-modes**	0.8695	0.7273	0.1165
**Genetic k-modes**	0.6598	0.5529	0.0472

**Table 19:** The RE of the four algorithms on the Mushroom dataset.

Algorithms	RE		
	Best	Avg.	Std.
**ABC-K-Modes**	0.8910	0.8537	0.0993
**K-modes**	0.7927	0.5956	0.0686
**Fuzzy k-modes**	0.8269	0.6969	0.1007
**Genetic k-modes**	0.6433	0.5418	0.0508

**Table 20:** The RI of the four algorithms on the Mushroom dataset.

Algorithms	RI		
	Best	Avg.	Std.
**ABC-K-Modes**	0.8114	0.7740	0.0914
**K-modes**	0.6799	0.5291	0.0518
**Fuzzy k-modes**	0.7212	0.6015	0.0796
**Genetic k-modes**	0.5443	0.5089	0.0144

Dermatology dataset has 366 data objects, each of which is described 34 categorical attributes. In terms of the class attribute, each data object belongs to one of the six classes: psoriasis, seborrheic dermatitis, lichen planus, pityriasis rosea, chronic dermatitis, and pityriasis rubra pilaris. Tables 21–24 summarize the comparison of the clustering results of ABC-K-Modes and the other three well-known algorithms on the Dermatology dataset according to *AC*, *PR*, *RE*, and *RI*, respectively.

**Table 21:** The AC of the four algorithms on the Dermatology dataset.

Algorithms	AC		
	Best	Avg.	Std.
**ABC-K-Modes**	0.8361	0.7652	0.0339
**K-modes**	0.7951	0.6984	0.0752
**Fuzzy k-modes**	0.7240	0.6848	0.0449
**Genetic k-modes**	0.7404	0.6246	0.0693

**Table 22:** The PR of the four algorithms on the Dermatology dataset.

Algorithms	PR		
	Best	Avg.	Std.
**ABC-K-Modes**	0.8961	0.8221	0.0538
**K-modes**	0.8866	0.7742	0.0795
**Fuzzy k-modes**	0.8205	0.7322	0.0609
**Genetic k-modes**	0.7294	0.6918	0.0645

**Table 23:** The RE of the four algorithms on the Dermatology dataset.

Algorithms	RE		
	Best	Avg.	Std.
**ABC-K-Modes**	0.7620	0.6508	0.0570
**K-modes**	0.7316	0.5716	0.0796
**Fuzzy k-modes**	0.6660	0.5661	0.0527
**Genetic k-modes**	0.6358	0.5185	0.0539

**Table 24:** The RI of the four algorithms on the Dermatology dataset.

Algorithms	RI		
	Best	Avg.	Std.
**ABC-K-Modes**	0.9073	0.8548	0.0255
**K-modes**	0.8882	0.8206	0.0503
**Fuzzy k-modes**	0.8630	0.8274	0.0297
**Genetic k-modes**	0.8801	0.8293	0.0257

From the experimental results shown in Tables 1–24, we can see that our proposed ABC-K-Modes achieves higher Best, Avg., and lower Std. values in *AC*, *PR*, *RE*, and *RI* in most cases, and therefore ABC-K-Modes in general outperforms the other three algorithms in terms of *AC*, *PR*, *RE*, and *RI*, respectively. The reason for the success of ABC-K-Modes is due to its effective combination of global search (exploration) and local search (exploitation). This is achieved by the adoption of the OKM operator and the ABC optimisation framework. Therefore, the proposed ABC-K-Modes can obtain optimal or near optimal results. In Table 25, we list the average running time over twenty trials for the proposed ABC-K-Modes and the other three popular algorithms on the six different datasets. The results in Table 25 show that the size/dimension has a direct effect on the running time of these four algorithms. Specifically, the larger the size/dimension of data set is, the more time it is for these algorithms to find the satisfactory solution. This is consistent with the analysis of time complexity in the complexity analysis section. Compared to the k-modes algorithm, the ABC-K-Modes takes more time to execute due to the introduction of the ABC optimization strategy. However, we also notice that the running time difference between our ABC-K-Modes approach and traditional K-Modes approach decreases with the increase of the size and dimensions of the dataset, and this seems promising. For instance, for the mushroom dataset, which contains the largest number of records, the performance of ABC-K-Modes is closest to that of K-Modes compared to the situation on the other datasets. Finally, we will further explore the acceleration issue of the ABC-K-Modes in our future work.

**Table 25:** The average running time of the four algorithms on different datasets.

Datasets (number of data objects, number of attributes)	Average running time (seconds)			
	ABC-K-Modes	K-Modes	Fuzzy K-Modes	Genetic K-Modes
**Zoo (101,17)**	11.3711	0.0237	0.0352	0.2774
**Breast Cancer (699,10)**	30.2426	0.3631	0.0441	9.1701
**Soybean dataset (47,36)**	6.4793	0.0129	0.0252	0.2059
**Lung Cancer (32,57)**	6.9895	0.0125	0.0256	0.1663
**Mushroom (8124,23)**	738.1470	90.2529	1.8341	3270.2254
**Dermatology (366,34)**	92.7335	0.2294	0.2330	6.6162

## Conclusions and Future Work

In real-world applications, data objects characterised by categorical attributes are frequently encountered. The k-modes type algorithms are well known for their high efficiency to clustering categorical data. However, it is acknowledged that this type of algorithms is prone to fall into local optima.

To address this issue, in this research we proposed a novel clustering algorithm ABC-K-Modes on the basis of the traditional k-modes algorithm and ABC optimisation procedure. In our algorithm, the search process of employee bees and onlookers is implemented by introducing a specific procedure named OKM, and the search process of scouts are performed by random exploration. To accelerate the convergence of the ABC-K-Modes, we adopt the idea of multi-source search for the search of scout bees. Moreover, we analysed the time and space complexity of the proposed algorithm ABC-K-Modes, and tested ABC-K-Modes on six real-world categorical datasets derived from the UCI Machine Learning Repository. The experimental results demonstrated that our proposed algorithm was superior to the other three well-known algorithms according to the evaluation measures *AC*, *PR*, *RE*, and *RI*, respectively.

In the near future, we will explore the acceleration issue of the ABC-K-Modes, and extend this approach to cluster mixed data containing both numeric and categorical attributes. We will investigate the potential of ABC-K-Modes when applied to social media data. Furthermore, we would also like to explore other swarm intelligent algorithms for clustering categorical data as well as mixed data.
